# Impact of Sex and Genetic Variation in Relevant Pharmacogenes on the Pharmacokinetics and Safety of Valsartan, Olmesartan and Hydrochlorothiazide

**DOI:** 10.3390/ijms242015265

**Published:** 2023-10-17

**Authors:** Paula Soria-Chacartegui, Pablo Zubiaur, Dolores Ochoa, Marcos Navares-Gómez, Houwaida Abbes, Gonzalo Villapalos-García, Alejandro de Miguel, Eva González-Iglesias, Andrea Rodríguez-Lopez, Gina Mejía-Abril, Samuel Martín-Vilchez, Sergio Luquero-Bueno, Manuel Román, Francisco Abad-Santos

**Affiliations:** 1 Clinical Pharmacology Department, Hospital Universitario de La Princesa, Faculty of Medicine, Instituto de Investigación Sanitaria La Princesa (IP), Universidad Autónoma de Madrid (UAM), 28006 Madrid, Spain; 2 Biochemistry Department, LR12SP11, Sahloul University Hospital, 4011 Sousse, Tunisia; 3 Faculty of Pharmacy of Monastir, University of Monastir, 5019 Monastir, Tunisia; 4 Centro de Investigación Biomédica en Red de Enfermedades Hepáticas y Digestivas (CIBERehd), Instituto de Salud Carlos III, 28029 Madrid, Spain

**Keywords:** valsartan, olmesartan, hydrochlorothiazide, pharmacogenetics, *ABCB1* and *SLC22A1*

## Abstract

Drug combination therapy is the most common pharmacological strategy for hypertension management. No pharmacogenetic biomarkers for guiding hypertension pharmacotherapy are available to date. The study population were 64 volunteers from seven bioequivalence trials investigating formulations with valsartan, olmesartan and/or hydrochlorothiazide. Every volunteer was genotyped for 10 genetic variants in different transporters’ genes. Additionally, valsartan-treated volunteers were genotyped for 29 genetic variants in genes encoding for different metabolizing enzymes. Variability in pharmacokinetic parameters such as maximum concentration (C_max_) and time to reach it (t_max_), the incidence of adverse drug reactions (ADRs) and blood pressure measurements were analyzed as a function of pharmacogenetic and demographic parameters. Individuals with the *ABCB1* rs1045642 T/T genotype were associated with a higher valsartan t_max_ compared to those with T/G and G/G genotypes (*p* < 0.001, β = 0.821, R^2^ = 0.459) and with a tendency toward a higher postural dizziness incidence (11.8% vs. 0%, *p* = 0.070). A higher hydrochlorothiazide dose/weight (DW)-corrected area under the curve (AUC_∞_/DW) was observed in *SLC22A1* rs34059508 G/A volunteers compared to G/G volunteers (*p* = 0.050, β = 1047.35, R^2^ = 0.051), and a tendency toward a higher postural dizziness incidence (50% vs. 1.6%, *p* = 0.063). Sex impacted valsartan and hydrochlorothiazide pharmacokinetics, showing a lower exposure in women, whereas no significant differences were found for olmesartan pharmacokinetics.

## 1. Introduction

Angiotensin II receptor blockers (ARBs), such as valsartan and olmesartan, are widely-used antihypertensive drugs. Their mechanism of action is based on the selective antagonism of the angiotensin II receptor type 1, which leads to an increase in renin, angiotensin I and angiotensin II plasmatic levels and a decrease in aldosterone plasmatic levels, causing a decrease in blood pressure (BP) [1,2]. While valsartan and olmesartan show a similar absolute oral bioavailability (approximately 25%), olmesartan is formulated as a pro-drug, olmesartan medoxomil, which is rapidly and completely converted to its active metabolite, olmesartan, by different esterases [1,2]. Valsartan and olmesartan are highly bound to plasmatic proteins (99%) and the maximum plasma concentration (C_max_) of both drugs is reached approximately 2 h after drug administration (t_max_). Valsartan is slightly metabolized to an inactive compound by unknown enzymes and it is excreted mainly unaltered in urine and feces, with an elimination half-life (t_1/2_) of 6 h [1]. Olmesartan is not further metabolized; thus, it is excreted unaltered in urine and feces, with a t_1/2_ of approximately 13 h [2].

Diuretic thiazides, such as hydrochlorothiazide, are also used in high-BP (HBP) treatment. They increase urine production by the inhibition of sodium and chloride reabsorption, causing a BP reduction [3]. Hydrochlorothiazide absolute oral bioavailability is higher than olmesartan and valsartan (65–75%) and its t_max_ is variable (1 to 5 h). It is not metabolized; therefore, it is excreted unaltered in urine, with a t_1/2_ ranging from 5 to 15 h [3].

Antihypertensive treatment is commonly administered in a combination therapy in order to reach a higher efficacy with lower adverse drug reactions (ADRs) incidence, since a better BP control is achieved through the sum of the different mechanisms of action [4]. Pharmacogenetics is a useful tool for the development of personalized medicine, as it provides biomarkers to tailor treatment specifically for each patient, increasing efficacy and reducing the risk of ADRs. However, no pharmacogenetic recommendation for antihypertensive treatment is available. The aim of this research was to evaluate the impact of genetic variation in the *ABCB1*, *ABCC2*, *ABCG2*, *SLCO1B1* and *SLC22A1* transporters on valsartan, olmesartan and hydrochlorothiazide pharmacokinetics, safety and BP variability. Secondarily, the impact of genetic variation in the metabolizing enzymes of the cytochrome P450 family (CYP) *CYP2A6*, *CYP2B6*, *CYP2D6*, *CYP2C8*, *CYP2C9*, *CYP2C19*, *CYP3A4*, *CYP3A5* and *UGT1A1* on valsartan pharmacokinetics was analyzed. The present work is part of the La Princesa Multidisciplinary Initiative for the Implementation of Pharmacogenetics (PriME-PGx) [5].

## 2. Results

**Figure 1:**
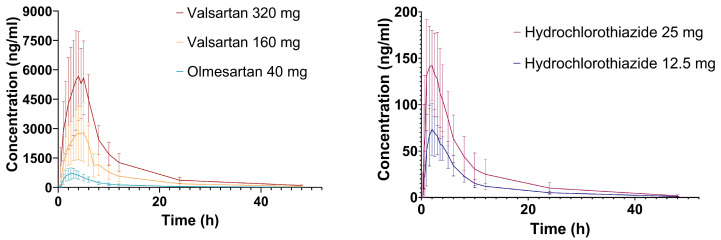
Time–concentration curves for valsartan, olmesartan and hydrochlorothiazide. Data are shown as mean ± standard deviation. The mean minus standard deviation negative values are not plotted.

Of the 212 volunteers participating in the seven bioequivalence clinical trials (CTs), 64 (27 women and 37 men) gave written informed consent for the candidate gene study and had previously been genotyped in other research [6]. Women were older than men and presented a lower height, weight and body mass index (BMI) (*p* = 0.011, *p* < 0.001, *p* < 0.001, *p* = 0.029, respectively). No differences were found between individuals self-reported as Europeans and Latin-Americans or Africans (since only one volunteer was self-identified as African, it was merged in the Latin-American group, which was named “Other”) or among CTs (Table 1). Since hydrochlorothiazide was present in all formulations but CT 1 (n = 6), pharmacokinetic data for this drug were available in 58 healthy volunteers; olmesartan data were available in 37 and valsartan in 26. Amlodipine pharmacokinetics and safety derived from these healthy volunteers were evaluated in an association study previously published [6]. The time–concentration curves for the three drugs included in this study are shown in Figure 1.

### 2.1. Hydrochlorothiazide

For hydrochlorothiazide 12.5 mg, the mean area under the curve extrapolated to infinity (AUC_∞_) and C_max_ were 593.51 ± 133.37 ng*h/mL and 87.67 ± 24.59 ng/mL; and 1203.68 ± 396.52 ng*h/mL and 166.19 ± 48.31 ng/mL for the 25 mg dose, showing linear pharmacokinetics. No significant differences in t_max_ and t_1/2_ were found between doses. Hydrochlorothiazide’s dose-corrected AUC_∞_ (AUC_∞_/D) was significantly higher in women compared to men (58.42 ± 13.45 ng*h/mL*mg versus 40.75 ± 6.02 ng*h/mL*mg, *p* < 0.001) and t_1/2_ was significantly lower (univariate *p*-value (*p*_uv_) = 0.006, multivariate *p*-value (*p*_mv_) = 0.008, β = −1.014, R^2^ = 0.103), with no differences in dose-corrected C_max_ (C_max_/D) (6.97 ± 2.19 versus 6.79 ± 1.80 ng/mL*mg) or in dose/weight-corrected AUC_∞_ (AUC_∞_/DW), dose/weight-corrected C_max_ (C_max_/DW) and t_max_ (Table 2). No pharmacokinetics differences according to CT or biogeographic origin were observed (Table S1). Higher hydrochlorothiazide AUC_∞_/DW was observed in *SLC22A1* rs34059508 G/A volunteers compared to G/G volunteers (*p*_uv_ = 0.031, *p*_mv_ = 0.050, β = 1047.35, R^2^ = 0.051) (Table 2). No significant differences were found for the remaining genotypes or phenotypes (Table S1).

### 2.2. Valsartan

For valsartan 160 mg, the mean AUC_∞_ and C_max_ were 27,359.24 ± 12,523.54 ng*h/mL and 3452.35 ± 1485.89 ng/mL; and 54,443.93 ± 13,513.52 ng*h/mL and 6623.88 ± 1653.98 ng/mL for the 320 mg strength, showing linear pharmacokinetics. No significant differences in t_max_ and t_1/2_ were found between doses. Valsartan AUC_∞_/DW was significantly lower in women compared to men (*p*_uv_ = 0.023), with no differences in AUC_∞_/D (166.73 ± 79.11 versus 176.19 ± 53.34 ng*h/mL*mg), in C_max_/D (22.47 ± 9.48 versus 19.72 ± 5.92 ng/mL*mg) or in the remaining pharmacokinetic parameters (Table 3). No pharmacokinetic differences among CTs or biogeographic origins were observed (Table S2). A higher valsartan t_max_ was observed in *ABCB1* rs1128503 T/T individuals compared to T/C and to C/C individuals (*p*_uv_ = 0.001 and *p*_uv_ = 0.009, respectively) and in *ABCB1* rs1045642 T/T individuals compared to T/C and to C/C individuals (*p*_uv_ = 0.018 and *p*_uv_ = 0.012, respectively, *p*_mv_ < 0.001, β = 0.821, R^2^ = 0.459) (Table 3). *ABCB1* rs2032582 T/T individuals showed higher valsartan t_max_ than T/G+G/A+G/G individuals (*p*_uv_ = 0.020) (Table 3). Moreover, valsartan t_max_ was significantly higher in CYP3A5 intermediate metabolizers (IMs) compared to poor metabolizers (PMs) (*p*_uv_ = 0.014). However, differences disappeared in the multivariate analysis. CYP2D6 PMs showed higher valsartan t_1/2_ compared to IMs, to normal metabolizers (NMs) and to ultrarrapid metabolizers (UMs) (*p*_uv_ = 0.015, *p*_uv_ = 0.026 and *p*_uv_ = 0.016, *p*_mv_ = 0.002, β = −5.005, R^2^ = 0.342) (Table 3). Additionally, volunteers with a SLCO1B1 poor function (PF) or decreased function (DF) phenotype showed a tendency toward higher valsartan AUC_∞_/DW (15,092.84 ± 3825.96 versus 10,583.92 ± 4341.31 ng*h*kg/mL*mg, *p*_uv_ = 0.066) and C_max_/DW (1749.98 ± 118.10 versus 1309.98 ± 481.01 ng*kg/mL*mg, *p*_uv_ = 0.087) compared to normal function (NF) volunteers (Table S2).

### 2.3. Olmesartan

The mean AUC_∞_ and C_max_ for olmesartan 40 mg were 5879.71 ± 1813.67 ng*h/mL and 775.16 ± 255.19 ng/mL. AUC_∞_ and C_max_ were significantly higher in women compared to men (184.38 ± 48.92 versus 134.07 ± 39.54 ng*h/mL*mg, *p* = 0.002 and 23.25 ± 5.01 versus 18.03 ± 6.45 ng/mL*mg, *p* = 0.019, respectively), with no differences after DW adjustment (Table 4). In CT 5 (notably, the only CT with olmesartan with a hydrochlorothiazide dose of 25 mg, compared to 12.5 mg in CT 6 and 7), olmesartan t_max_ was lower compared to the other CTs (*p*_uv_ = 0.006) (Table 4). No differences in pharmacokinetic parameters according to biogeographic origin, genotypes or phenotypes were found (Table S3). A tendency toward a higher olmesartan C_max_/DW was observed in volunteers with SLCO1B1 PF+DF phenotypes compared to those with the NF phenotype (1679.92 ± 517.46 versus 1358.67 ± 374.76 ng*kg/mL*mg, *p*_uv_ = 0.066) (Table S3).

### 2.4. Blood Pressure and Heart Rate

**Figure 2:**
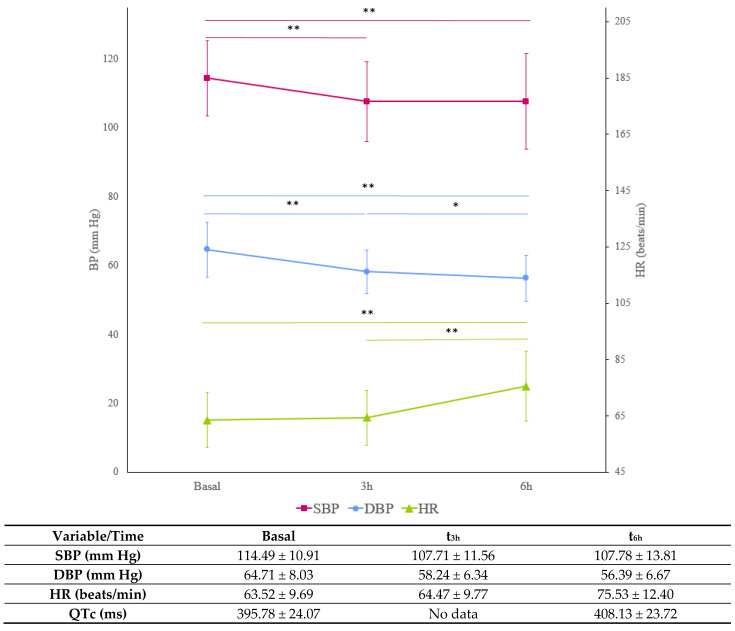
Systolic blood pressure (SBP), diastolic blood pressure (DBP), heart rate (HR) and QTc in basal state, t_3h_ and t_6h_. Data are shown as mean ± standard deviation. **: *p* < 0.001, *: *p* < 0.05.

Systolic and diastolic BP (SBP and DBP, respectively) significantly decreased at t_3h_ and t_6h_ after drug intake, whereas heart rate (HR) significantly increased at t_6h_ (n = 64, Figure 2). The QT interval corrected by HR (QTc) was prolonged by 12.5 ms at t_6h_ compared to the basal situation (408.13 ± 23.72 ms and 395.78 ± 24.07, respectively, *p* < 0.001). The SBP reduction at t_6h_ was significantly higher in women compared to men (−10.91 ± 10.65 versus −3.57 ± 11.24, respectively, *p* = 0.011). The HR increase at t_6h_ was significantly higher in those CTs with olmesartan (CT 5, 6 and 7: 16.46 ± 11.49 beats/min) compared to CTs with valsartan (CT 1: 1.92 ± 5.44 beats/min, *p* = 0.001; CT 2, 3 and 4:6.80 ± 8.25 beats/min, *p* = 0.001). No further significant differences in SBP, DBP, HR or QTc changes were found according to sex, biogeographic origin, CTs, SLCO1B1 or ABCG2 phenotype or *ABCB1*, *ABCC2* and *SLC22A1* genotypes.

### 2.5. Adverse Drug Reactions

A total of 36 ADRs in 28 healthy volunteers were observed. A 45% higher valsartan AUC_∞_ was observed in individuals experiencing ADRs compared to those volunteers without ADRs (44,902.94 ± 20,396.02 versus 30,817.13 ± 14,857.38 ng*h/mL, respectively, *p* = 0.054), with no differences in olmesartan and hydrochlorothiazide pharmacokinetics.

The most common ADR was headache (21 out of 64, 32.81%), followed by dizziness (3 out of 64, 4.69%), postural dizziness and nausea (2 out of 64, 3.13% for both). ADRs occurred in 28 out of the 58 (48.3%) individuals treated with triple pharmacotherapy, whereas none of the six individuals treated with double pharmacotherapy suffered from an ADR (0.0%, *p* = 0.031). A tendency toward a higher postural dizziness incidence was observed in *ABCB1* rs1045642 T/T volunteers (2 out of 17, 11.8%) compared to T/C and C/C volunteers (0 out of 22 and 24, respectively, 0% and 0%, *p* = 0.070) and in *SLC22A1* rs34059508 G/A individuals (1 out of 2, 50%) compared to G/G individuals (1 out of 61, 1.6%, *p* = 0.063). No further differences in ADR incidence were found according to sex, biogeographic origin, CTs or the remaining transporter genotypes.

## 3. Discussion

Due to the high incidence of HBP globally and the poor success rate of antihypertensive treatment, co-administration of two or three antihypertensive drugs is frequent [7]. Pharmacogenetics could play an important role in drug combination selection, allowing the individualized prescription of effective and safe antihypertensive drugs, increasing drug adherence and patient quality of life. To date, limited research has been published on the pharmacogenetics of the three drugs analyzed in this investigation, which prompted this exploratory candidate gene study.

The pharmacokinetic parameters for the three drugs analyzed in this research were consistent with the information available in the literature [1,3,8,9,10,11,12]. Regarding valsartan and hydrochlorothiazide, a clear linear dose-dependent relationship was observed; duplicating the dose was associated with a two-fold increase in AUC and C_max_, as has been described in previous works [3,8]. To our knowledge, this is the first study to find lower valsartan exposure in women after DW adjustment. As valsartan is a lipophilic drug [13,14], its tissue distribution is expected to be higher in women, who usually have a higher body fat percentage [15], which may cause the observed lower plasmatic concentration. However, the differences disappeared in the multivariate analysis; thus, further research is needed to dismiss whether these differences are an artefact caused by the interaction of other variables not evenly distributed by sex. For hydrochlorothiazide, a lower t_1/2_ was observed in women compared to men. However, men and women’s t_1/2_ values fitted in the range established in the drug label for fasting volunteers, no differences in AUC_∞_/DW were observed and AUC_∞_/D was higher in women due to their lower weight [3]. A higher elimination rate in women was described in previous works with other drugs [16,17]; however, this was not the case for hydrochlorothiazide [18]. Due to the novelty of these results, further research is warranted to clarify the impact of sex on antihypertensive treatment.

When olmesartan was co-administered with a 25 mg hydrochlorothiazide dose (CT 5), C_max_ was reached faster than when the hydrochlorothiazide dose was 12.5 mg (CT 6 and 7). Thus, hydrochlorothiazide may interact with transporters involved in olmesartan pharmacokinetics, accelerating its absorption. However, no previous information of this interaction is available in the literature and other factors might be involved, such as differences in the drug formulation.

As expected, antihypertensive treatment resulted in a decrease in SBP and DBP, which might explain some of the ADRs observed (i.e., headache or dizziness) [19]. In fact, DBP was slightly below the normal range (60–80 mm Hg) at t_3h_ and t_6h_ (58.24 ± 6.34 and 56.39 ± 6.67 mm Hg, respectively). The SBP reduction was higher in women compared to men, as reported in other studies [20], with no differences according to the administered drug combination, genotypes or phenotypes. However, olmesartan and hydrochlorothiazide exposure may be responsible for these differences, as women presented higher AUC_∞_/D values of these two drugs compared to men. Thus, none of these genetic variants seem to be useful predictors of antihypertensive treatment efficacy, whereas olmesartan and hydrochlorothiazide exposure might be a useful predictor of BP reduction. Therefore, therapeutic drug monitoring may be a useful tool for the management of at-risk patients. However, as these results derive from healthy volunteers, further research in patients should be conducted. Regarding safety, the ADRs suffered were consistent with the information of the three drugs’ labels [1,2,3]. A HR increase was observed, consistent with the BP decrease, possibly caused by the baroreceptor reflex; however, values were maintained within the normal range, and treatment safety was therefore not compromised [21]. The different drug and dose combinations were well tolerated, with no significant differences in ADR incidence among them, as previously reported [22]. In contrast, valsartan AUC_∞_ was related to ADR incidence but not that of olmesartan and hydrochlorothiazide, which suggests that the valsartan tolerability profile may be slightly inferior when pharmacotherapy is initiated. Further studies with HBP patients and multiple dose administration are needed to check whether the associations between olmesartan, hydrochlorothiazide and valsartan exposure, efficacy and ADR incidence might be useful in clinical practice.

Valsartan and hydrochlorothiazide pharmacokinetics were altered by *ABCB1* and *SLC22A1* genetic variation, respectively. *ABCB1* codes for the P-glycoprotein (P-gp) transporter, located in enterocytes, renal cells, hepatocytes and blood–brain barrier cells [23]. In this study, having the reference genotype (T/T) in any of the three *ABCB1* variants analyzed (rs1045642 T>C, rs1128503 T>C and rs2032582 T>G/A) slowed valsartan absorption. Our findings hence suggest that valsartan is a P-gp substrate, like other drugs of the ARB family, such as losartan or candesartan [23,24]. In this research, the association between t_max_ and the *ABCB1* rs1045642 T/T genotype was reinforced by its association with a higher postural dizziness incidence. However, the individual impact of each genetic variant on P-gp expression, structure and/or function is unknown, and its study is hampered by the partial linkage disequilibrium between them and the different locations of the transporter [25]. Similarly, *SLC22A1* codifies for the hepatic organic cationic transporter 1 (OCT1), involved in drug uptake into hepatic cells [26]. Carriers of the *SLC22A1* rs34059508 G>A A allele, proposed as a decreased function variant [26], showed higher hydrochlorothiazide exposure and a tendency toward higher postural dizziness incidence, suggesting that this transporter is involved in hydrochlorothiazide hepatic uptake. To our knowledge, no evidence of this association is available in the literature; however, hydrochlorothiazide was previously described as substrate of other transporters of the SLC22A family [27]. Additionally, OCT1 was found to be involved in amlodipine transport in a previous work derived from the same cohort of healthy volunteers [6]. Thus, this transporter might be especially relevant in antihypertensive treatment. Nevertheless, these results should be considered cautiously and its potential clinical utility requires adequate prior characterization of both transporters.

For CYP2D6, an association toward a higher valsartan t_1/2_ in PMs compared to UMs, NMs and IMs was found, which suggests that this enzyme might be involved in its metabolism. However, no differences were found in exposure, probably because only 20% of valsartan is metabolized and only two CYP2D6 PMs were included in this research. CYP2D6 seem to participate in the metabolism of other antihypertensive drugs, such as diltiazem or amlodipine, as seen in our previous work derived from the same cohort of healthy volunteers [6]. Notwithstanding, its role on valsartan metabolism has not been described. Previous studies show contradictory results; while some propose CYP enzymes (and specifically CYP2C9) as being responsible for that 20% of valsartan metabolism [28,29], others propose that no CYP enzymes are involved [30]. In this research, no significant results were found for CYP2C9; however, only one PM was included, which showed exposure data similar to CYP2C9 NMs. For CYP3A5, the observed differences in t_max_ between PMs and IMs might be explained by the different proportion of *ABCB1* rs1128503 T/T volunteers within each phenotype group (2 out of 4 IMs were T/T [50.0%], 1 out of 21 PMs were T/T [4.8%], *p* = 0.057). Furthermore, the disappearance of this association in the multivariate analysis and the lack of differences in AUC_∞_/DW and C_max_/DW suggest that *CYP3A5* genetic variation might not have any impact on valsartan pharmacokinetics. Thus, our results suggest that CYP2D6 might be involved in valsartan metabolism and its genetic variation may affect its pharmacokinetics, whereas *CYP2C9* and *CYP3A5* genetic variation may be less relevant.

Lastly, although the threshold for significance was not reached, the SLCO1B1 phenotype seemed to alter valsartan and olmesartan exposure: the only valsartan-treated volunteer with a PF phenotype showed a 1.9- and 1.5-fold higher valsartan AUC_∞_/DW value compared to volunteers with the NF and DF phenotypes, respectively. For the only olmesartan-treated volunteer with a PF phenotype, minor differences were observed: a 1.3- and 1.1-fold higher AUC_∞_/DW value compared to volunteers with the NF and DF phenotypes. Consistently, valsartan and olmesartan were previously described as substrates of the transporter codified by *SLCO1B1*, the organic anion transporter polypeptide OATP1B1 [31,32,33,34]. These results may lay the groundwork in establishing an association between valsartan and olmesartan pharmacokinetics and SLCO1B1 phenotypes in the future, as *SLCO1B1* is a useful pharmacogenetic biomarker already used in treatment with other drugs, such as statins [35].

Nevertheless, further research with different drug combinations in less controlled conditions (e.g., food, smoking, other treatments) is needed to determine the usefulness of these associations in clinical practice. If replicated, these biomarkers may be implemented in HBP clinical management and may be used to reach a higher efficacy with lower ADRs incidence, increasing treatment adherence and reducing treatment discontinuation or treatment changes.

- LIMITATIONS

This research has two main limitations. Firstly, the administration of a single dose and the inclusion of healthy subjects do not allow us to conclude on valsartan, olmesartan and hydrochlorothiazide long-term safety, pharmacodynamics and pharmacokinetics. Secondly, the modest and arbitrary sample size available are limiting, since the candidate-gene study was conducted based on the available pharmacogenetic, pharmacokinetic and safety data from seven bioequivalence trials. The limited sample size reduces the statistical power and the chances to encounter biomarkers of interest with a low prevalence within the sample population, such as CYP2C9 PMs. Due to the absence or reduced olmesartan, hydrochlorothiazide and valsartan metabolism, the main candidate genes available were transporters, which are poorly characterized structurally (i.e., which genetic variants define each allele, such as *ABCB1*, *ABCC2*, *ABCG2* and *SLC22A1*) and functionally (i.e., the impact of alleles on the protein function, such as *ABCB1*, *ABCC2* and *SLC22A1*). In contrast, the present research strictly controlled confounding factors that affect pharmacokinetics, pharmacodynamics and safety in clinical settings with patients (e.g., diet conditions, concomitant use of other drugs, smoking, etc.).

## 4. Material and Methods

### 4.1. Study Design and Procedures

The data used in this research were obtained from seven CTs performed at the Clinical Trials Unit of Hospital Universitario de La Princesa (UECHUP), Madrid (Spain), from 2012 to 2018 (Table 5). In every CT, the test (T) and reference (R) formulations were film-coated tablets that contained at least one of the antihypertensive drugs studied (Table 5). The R formulation administered was Exforge^®^ (Novartis Europharm Limited, London, UK) for CT 1, Exforge HCT^®^ (Novartis Europharm Limited, London, UK) for CTs 2, 3 and 4, Sevikar HCT^®^ (Daiichi Sankyo Spain, S.A) for CT 5 and 6 and Olmetec Plus^®^ (Daiichi Sankyo Spain, S.A, Madrid, Spain) and Norvas^®^ (Pfizer Inc., New York City, NY, USA) for CT 7, all of them at the same dose as the T formulation. In all of the CTs, a single oral dose of each drug was administered under fasting conditions; they were phase I, open-label, single-center, crossover and randomized CTs, with two sequences and two periods for olmesartan and hydrochlorothiazide, and four sequences and four periods for valsartan (replicated design). In each period, volunteers were admitted at UECHUP at 10 pm (day 0) and remained there for at least 12 h after drug administration at 9 am of Day 1. The wash out time for CT 1, 2, 3 and 4 was 14 days between Period 1 and 2, while it was 7 days between Periods 2, 3 and 4. For CTs 5, 6, and 7, the wash out time was 21 days. All the formulations included a 10 mg amlodipine dose and its pharmacogenetic results are already published [6]. Although amlodipine is a widely-used and effective antihypertensive drug, the dose administered was the same in all formulations; therefore, it could be discarded as being responsible for the observed differences in efficacy and safety.

During hospital admission and at additional visits in each period, at least 17 blood samples were extracted from pre-dose to 48 h after drug administration. The blood samples obtained were used for the plasmatic quantification of drug levels, which was conducted in an external laboratory by high-performance liquid chromatography coupled to tandem mass spectrometry. For the blinded plasmatic concentration determination, a method validated according to the European Medicines Agency (EMA) guidelines was used. The lower limit of quantification was established at 1.01 ng/mL for hydrochlorothiazide, 2.51 ng/mL for olmesartan and 20.02 ng/mL for valsartan.

Inclusion criteria comprised: women or men between 18 and 55 years old, free from organic or psychological disorders, with normal medical records, physical examination, vital signs, electrocardiogram (ECG) and laboratory tests. Exclusion criteria comprised: having aBMI outside the 18.5–30.0 kg/m^2^ range, being pregnant or breastfeeding women, smoking or alcoholism, positive drug screening, blood donation in the previous month, inability to collaborate or follow instructions and participation in other studies with investigational drugs in the previous three months. The CT protocols were revised and approved by the Hospital’s Research Ethics Committee and by the Spanish Drugs Agency (AEMPS). CT and data handling were performed complying with the Good Clinical Practice guidelines [36], the Declaration of Helsinki [37] and the current Spanish legislation regarding clinical trials. All volunteers provided an additional informed consent form to participate in the present pharmacogenetic study. The demographic information obtained from the CTs included sex, age, weight, height, BMI and biogeographic origin and was self-reported by the volunteers.

### 4.2. Pharmacokinetics, Safety and Pharmacodynamics

C_max_ and t_max_ were directly retrieved from the plasmatic time–concentration curves. The remaining parameters were estimated with WinNonlin Professional Edition version 2 (CT 1) or version 7 (CT 2-7) (Scientific Consulting, Inc., Cary, NC, USA) using a non-compartmental approach. The area under the time–concentration curve (AUC) from pre-dose to the last observed concentration time (48 h) (AUC_t_) was calculated according to the trapezoidal rule. For AUC extrapolation to infinity (AUC_∞_), AUC_t→∞_ was calculated as C_t_/K_e_ (where C_t_ is the last detectable concentration and K_e_, the elimination slope) and added to AUC_t_. Finally, t_1/2_ was calculated as −ln2/K_e_. For valsartan and hydrochlorothiazide, the T formulation was demonstrated to be bioequivalent to the R formulation in every CT. Hence, the arithmetic mean of the pharmacokinetic parameters of both formulations was calculated for each volunteer. For olmesartan, not all T formulations were demonstrated to be bioequivalent to the R formulation; therefore, only the pharmacokinetic parameters of the R formulation were used for the analysis.

For safety assessment, adverse event (AE) identification was performed by direct question to the volunteers or by spontaneous notification. The algorithm of Spanish pharmacovigilance system [38] was used to address AE causality: only AEs with a definite, probable or possible causality were considered ADRs and considered for the statistical analysis. Additionally, BP and HR measurements were scheduled before, 2 to 3 h (t_3h_) and 5 to 6 h (t_6h_) after drug intake and ECGs were scheduled before and 5 to 6 h (t_6h_) after drug intake. HR and QTc prolongation measurements were used for the safety analysis. Since this candidate gene study is focused on antihypertensive drugs, BP measurements were used for the pharmacodynamics analysis.

### 4.3. Genotyping and Phenotyping

Blood samples collected in EDTA-K2 tubes during the CTs were used for DNA extraction with a MagNA Pure instrument (Roche Applied Science, Branford, CT, USA) or a Maxwell^®^ RSC Automated DNA extractor (Promega Biotech Iberica S.L, Madrid, Spain). Volunteers were genotyped for 39 variants located in 14 pharmacogenes in a QuantStudio 12 K Flex qPCR instrument with an OpenArray thermal block (Applied Biosystems, Thermofisher, Waltham, MA, USA) using the PriME-PGx Very Important Pharmacogene Open Array (VIPOA) custom genotyping array, version VIPOA1, VIPOA2 or VIPOA3 (Table 6). For valsartan, variants in transporters (*ABCB1*, *ABCC2*, *ABGC2*, *SLC22A1* and *SLCO1B1*) and metabolizing enzymes (*CYP2A6*, *CYP2B6*, *CYP2C8*, *CYP2C9*, *CYP2C19*, *CYP2D6*, *CYP3A4*, *CYP3A5* and *UGT1A1*) were analyzed. For olmesartan and hydrochlorothiazide, only variants in *ABCB1*, *ABCC2*, *ABGC2*, *SLC22A1* and *SLCO1B1* transporters were included in the analysis, as these drugs are not metabolized. *CYP2D6* deletion (*5), duplication and the presence of hybrid structures were analyzed using two TaqMan^®^ copy number variation assays targeting exon 9 (Assay ID: Hs00010001_cn) and intron 2 (Assay ID: Hs04083572_cn) (Applied Biosystems, Foster City, CA, USA), as previously described [6]. For genotyping, star alleles were defined in accordance with PharmVar published definitions (available at: https://www.pharmvar.org (accessed on 1 August 2023)). Genotype information was translated into phenotype according to the Clinical Pharmacogenetics Implementation Consortium (CPIC) guidelines for *CYP2B6* [39], *CYP2C19* [40], *CYP2C9* [41], *CYP2D6* [42], *CYP3A5* [43], *SLCO1B1* [44] and *UGT1A1* [45], in accordance with PharmGKB/CPIC/PharmVar PGx gene-specific information tables, available at https://www.pharmgkb.org/page/pgxGeneRef (accessed on 1 August 2023), and the Dutch Pharmacogenetic Working Group (DPWG) guideline for *CYP3A4* [46]. For CYP2C8, phenotype was inferred as previously described [47].

### 4.4. Statistical Analysis

SPSS software (version 23, SPSS Inc., Chicago, IL, USA) was used to perform the statistical analysis. AUC_∞_ and C_max_ were divided by the dose/weight ratio (DW) to correct dose and weight effect on bioavailability. The Shapiro–Wilk test was applied for checking normality in variable distributions. For those variables not following a normal distribution, a logarithmic transformation was applied and normality was re-analyzed. Firstly, in the univariate analysis, pharmacokinetic parameters were analyzed according to sex, biogeographic origin, CT, genotypes and phenotypes. T-tests or ANOVA tests followed by a Bonferroni post-hoc were performed for the comparison of means for variables following a normal distribution with two or with three or more categories, respectively. For those variables not normally distributed, nonparametric tests were used: a Mann–Whitney test or a Kruskal–Wallis test, respectively. The multivariate analysis was performed by means of linear regression, in which those independent variables significantly related to the dependent variable in the univariate analysis (i.e., with *p*-values (*p*_uv_) lower than 0.05) were included. The multivariate *p*-value (*p*_mv_), the non-standardized β coefficient (β) and R^2^ are shown for significant associations. To analyze the effect of treatment on SBP and DBP, QTc interval and HR, paired sample T-tests were performed. Furthermore, ADR incidence was analyzed according to sex, biogeographic origin, CT and genotype or genotype-informed phenotypes by Chi^2^ or Fisher exact tests, when appropriate.

## 5. Conclusions

Valsartan and hydrochlorothiazide are proposed to be P-gp and OCT1 substrates, respectively, as *ABCB1* and *SLC22A1* genetic variation affect drug exposure and safety, and CYP2D6 may be involved in valsartan metabolism. However, the use of *ABCB1* and *SLC22A1* as pharmacogenetic biomarkers in clinical practice requires an improved characterization of both genes and the replication of these associations in independent additional clinical contexts. Furthermore, valsartan and olmesartan might be OATP1B1 substrates and their exposure may be conditioned by the *SLCO1B1* genotype. To the best of our knowledge, this is the first work to propose genetic variation in transporters as biomarker for valsartan, olmesartan and hydrochlorothiazide pharmacokinetics. Further research with an increased sample size is needed to confirm the relevance of the observed associations.

## Figures and Tables

**Table 1:** Demographic characteristics according to sex, biogeographic origin or CT.

Variable	n	Age (Years)	Height (m)	Weight (kg)	BMI (kg/m^2^)
Sex					
Men	37	24.57 (4.39)	1.78 (0.08)	78.77 (10.38)	24.89 (3.01)
Women	27	31.15 (10.69) *	1.62 (0.05) *	60.56 (7.57) *	23.21 (2.89) *
Biogeographic origin					
European	43	26.16 (7.35)	1.72 (0.10)	71.02 (11.97)	23.84 (3.08)
Other ^#^	21	29.76 (9.72)	1.68 (0.12)	71.21 (15.04)	24.89 (2.95)
CT					
1	6	22.17 (2.48)	1.73 (0.12)	65.83 (7.16)	22.11 (2.73)
2	4	30.00 (8.76)	1.70 (0.16)	77.93 (14.58)	26.85 (2.45)
3	8	27.25 (7.72)	1.68 (0.10)	63.50 (13.41)	22.23 (2.68)
4	8	26.13 (10.33)	1.68 (0.10)	64.96 (12.34)	22.93 (2.34)
5	12	30.58 (11.70)	1.73 (0.10)	76.54 (12.17)	25.65 (2.93)
6	10	28.40 (9.35)	1.72 (0.09)	73.50 (12.83)	24.86 (2.98)
7	16	26.19 (4.18)	1.72 (0.12)	72.60 (13.29)	24.36 (3.06)
Total	64	27.34 (8.30)	1.71 (0.11)	71.09 (12.94)	24.18 (3.05)

Data are shown as the mean (standard deviation). BMI: body mass index. CT: clinical trial. ^#^: individuals who self-reported as Latin-American (n = 20) or African (n = 1). *: *p* < 0.05 compared to men.

**Table 2:** Statistically significant associations between demographic and genetic variables and hydrochlorothiazide pharmacokinetic parameters.

Variable	n	AUC_∞_/DW (ng*h*kg/mL*mg)	C_max_/DW (ng*kg/mL*mg)	t_max_ (h)	t_1/2_ (h)
Sex					
Men	35	3213.59 (584.71)	465.31 (109.02)	1.89 (0.61)	9.70 (1.39)
Women	23	3508.15 (911.76)	491.54 (104.02)	2.04 (0.84)	8.66 (1.30) *
*SLC22A1* rs34059508					
G/G	55	3296.92 (731.93)	474.71 (107.74)	1.90 (0.67)	9.29 (1.46)
G/A	2	4344.26 (190.17) *	553.98 (48.15)	2.50 (0.71)	9.78 (0.81)

Data are shown as the mean (standard deviation). AUC_∞_/DW: dose-weight corrected area under the curve. C_max_/DW: dose-weight corrected maximum plasmatic concentration. t_1/2_: half-life. *: *univariate p-value* (*p*_uv_) < 0.05 compared to men or *SLC22A1* rs34059508 G/G genotype. Underlined: *multivariate p-value* (*p*_mv_) < 0.05 compared to men or *SLC22A1* rs34059508 G/G genotype.

**Table 3:** Statistically significant associations between demographic and genetic variables and valsartan pharmacokinetic parameters.

Variable	n	AUC_∞_/DW (ng*h*kg/mL*mg)	C_max_/DW (ng*kg/mL*mg)	t_max_ (h)	t_1/2_ (h)
Sex					
Men	11	13,374.37 (3839.37)	1490.04 (382.23)	3.37 (0.78)	8.80 (1.50)
Women	15	9658.52 (4290.87) *	1303.44 (509.40)	3.61 (1.03)	8.64 (2.75)
*ABCB1* rs1128503					
T/T	3	13,196.33 (7954.48)	1526.29 (916.55)	5.00 (0.25) *	9.06 (2.82)
T/C	10	12,529.81 (3854.25)	1449.58 (317.14)	3.52 (0.58)	9.89 (2.51)
C/C	12	9812.22 (4005.06)	1286.24 (478.30)	3.02 (0.80)	7.74 (1.62)
*ABCB1* rs1045642					
T/T	5	12,816.62 (5724.14)	1554.04 (672.27)	4.50 (0.88) *	8.56 (2.18)
T/C	9	11,536.06 (4803.69)	1322.30 (381.24)	3.13 (0.57)	9.96 (2.59)
C/C	11	10,429.64 (3912.43)	1348.96 (464.47)	3.25 (0.84)	7.86 (1.79)
*ABCB1* rs2032582					
T/T	4	12,627.06 (6593.84)	1481.92 (753.60)	4.50 (1.02) *	8.95 (2.32)
T/G	10	11,455.73 (4429.94)	1414.61 (367.05)	3.39 (0.81)	9.16 (2.59)
G/A	2	11,798.75 (6567.18)	1634.43 (628.50)	3.88 (0.35)	6.50 (1.24)
G/G	9	10,987.88 (3298.50)	1321.33 (410.01)	3.23 (0.86)	8.72 (2.16)
CYP2D6					
UM	2	11,533.20 (393.31)	1609.28 (269.46)	4.44 (0.44)	6.84 (1.63)
NM	11	12,329.25 (5352.08)	1462.70 (503.56)	3.65 (0.99)	8.70 (2.19)
IM	9	10,298.34 (4685.46)	1271.91 (531.72)	3.09 (0.85)	8.23 (1.50)
PM	2	10,170.64 (1857.41)	1202.63 (316.61)	3.32 (0.09)	13.43 (2.23) *
CYP3A5					
IM	4	11,497.08 (4442.61)	1439.20 (550.88)	4.44 (0.71) *	7.35 (1.34)
PM	21	11,268.83 (4635.61)	1369.18 (468.67)	3.27 (0.82)	9.03 (2.37)

Data shown as the mean (standard deviation). AUC_∞_/DW: dose-weight corrected area under the curve. C_max_/DW: dose-weight corrected maximum plasmatic concentration. t_1/2_: half-life. UM: ultrarrapid metabolizer. NM: normal metabolizer. IM: intermediate metabolizer. PM: poor metabolizer. *: *univariate p-value* (*p*_uv_) < 0.05 compared to *ABCB1* rs1128503 T/C and to C/C; or to rs1045642 T/C and to C/C, to *ABCB1* rs2032582 T/G+G/A+G/G genotypes, to CYP2D6 UMs, to NMs and to IMs or to CYP3A5 PMs. Underlined: *multivariate p-value* (*p*_mv_) < 0.05 compared to *ABCB1* rs1045642 T/C + C/C genotypes or to CYP2D6 UMs + NMs + IMs.

**Table 4:** Sex and statistically significant associations between demographic variables and olmesartan pharmacokinetic parameters.

	n	AUC_∞_/DW (ng*h*kg/mL*mg)	C_max_/DW (ng*kg/mL*mg)	t_max_ (h)	t_1/2_ (h)
Sex					
Men	25	10,539.83 (3009.88)	1406.74 (454.05)	2.72 (1.16)	9.24 (1.44)
Women	12	11,494.44 (3398.77)	1445.91 (344.42)	2.58 (0.76)	8.56 (1.08)
CT					
5	12	10,960.25 (3810.09)	1457.24 (538.37)	1.96 (0.69) *	9.36 (1.58)
6	10	12,067.51 (3220.87)	1596.56 (363.70)	3.20 (0.92)	8.85 (1.54)
7	15	9948.73 (2262.70)	1271.12 (295.86)	2.90 (1.07)	8.86 (1.06)

Data shown as the mean (standard deviation). CT: clinical trial. AUC_∞_/DW: dose-weight corrected area under the curve. C_max_/DW: dose-weight corrected maximum plasmatic concentration. t_1/2_: half-life. *: *univariate p-value* (*p*_uv_) < 0.05 compared to CT6 and CT7.

**Table 5:** Overview of the clinical trials involved in this pharmacogenetic study.

CT	Eudra-CT	Test Formulation	Volunteers ^#^
1	2013-004147-23	Amlodipine/Valsartan 10/160 mg	6
2	2017-000547-40	Amlodipine/Valsartan/Hydrochlorothiazide 10/160/25 mg	4
3	2017-001716-10	Amlodipine/Valsartan/Hydrochlorothiazide 10/160/12.5 mg	8
4	2017-001757-14	Amlodipine/Valsartan/Hydrochlorothiazide 10/320/25 mg	8
5	2017-005024-25	Amlodipine/Olmesartan/Hydrochlorothiazide 10/40/25 mg	12
6	2018-001378-11	Amlodipine/Olmesartan/Hydrochlorothiazide 10/40/12.5 mg	10
7	2018-002075-18	Amlodipine/Olmesartan/Hydrochlorothiazide 10/40/12.5 mg	16

CT: clinical trial. **^#^**: Volunteers who gave written informed consent for the candidate gene study.

**Table 6:** Genotyped genetic variants and alleles in which those genetic variants are present.

Gene	Allele	Genetic Variants Present in the Allele	Frequency (European Population) ^$^	Frequency (Latin-American Population) ^$^
*ABCB1*	N/A	rs1045642 (T>C)	T: 51.8%, C: 48.2%	T: 42.8%, C: 57.2%
	N/A	rs2032582 (T>G/A)	T: 41.0%, G: 57.3%, A: 1.8%	T: 36.9%, G: 57.2%, A: 5.9%
	N/A	rs1128503 (T>C)	T: 41.6%, C: 58.4%	T: 40.3%, C: 59.7%
*ABCC2*	N/A	rs2273697 (G>A)	G: 79.6%, A: 20.4%	G: 84.1%, A: 15.9%
*ABCG2*	N/A	rs2231142 (C>A)	C: 89.6%, A: 10.4%	C: 77.6%, A: 22.4%
*SLC22A1*	N/A	rs12208357 (C>T)	C: 93.7%, T:6.3%	C: 98.0%, T: 2.0%
	N/A	rs34059508 (G>A)	G:98.0%, A: 2.0%	G: 98.1%, A: 1.9%
	N/A	rs72552763 (GAT>delGAT)	GAT: 81.6%, delGAT: 18.4%	GAT: 71.2%, delGAT: 28.8%
*SLCO1B1*	*5	rs4149056 (T>C)	<0.01%	2.0%
	*15	rs4149056 (T>C), rs2306283 (A>G)	15.0%	24.0%
	*37	rs2306283 (A>G)	25.3%	39.0%
*UGT1A1 ^#^*	*80	rs887829 (C>T)	31.4%	38.3%
*CYP2D6*	*3	rs35742686 (A>delA)	1.6%	0.7%
	*4	rs3892097 (G>A), rs1065852(C>T)	18.5%	12.0%
	*6	rs5030655 (T>delT)	1.1%	0.5%
	*7	rs5030867 (A>C)	<0.01%	<0.01%
	*8	rs5030865 (G>T)	<0.01%	<0.01%
	*9	rs5030656 (TCT>delTCT)	2.8%	1.6%
	*10	rs1065852 (C>T)	1.6%	2.6%
	*14	rs5030865 (G>A)	<0.01%	<0.01%
	*17	rs28371706 (C>T)	0.4%	2.3%
	*41	rs28371725 (G>A)	9.2%	5.1%
*CYP2A6*	*9	rs28399433 (T>G)	7.1%	7.1%
*CYP2B6*	*4	rs2279343 (A>G)	40.9%	10.6%
	*5	rs3211371 (C>T)	11.6%	3.8%
	*6	rs2279343(A>G), rs3745274 (G>T)	23.3%	21.2%
	*7	rs2279343 (A>G), rs3745274 (G>T), rs3211371 (C>T)	2.5%	0.8%
	*9	rs3745274 (G>T)	1.5%	7.3%
*CYP2C8*	*2	rs11572103 (A>T)	A: 99.6%, T: 0.4%	A: 98.8%, T: 1.2%
	*3	rs10509681 (A>G)	A: 88.2%, G: 11.8%	A: 90.1%, G: 9.9%
	*4	rs1058930 (C>G)	C: 94.2%, G:5.8%	C: 98.1%, G: 1.9%
*CYP2C9*	*2	rs1799853 (C>T)	12.7%	7.6%
	*3	rs1057910 (A>C)	7.6%	4.0%
*CYP2C19*	*2	rs4244285 (G>A)	14.7%	7.2%
	*3	rs4986893 (G>A)	0.2%	<0.01%
	*4	rs28399504 (A>G)	0.2%	<0.01%
	*17	rs12248560 (C>T)	21.5%	16.7%
*CYP3A4*	*2	rs55785340 (T>C)	T: >99.9% C: <0.01%	T: >99.9% C: <0.01%
	*6	rs46464389 (delA>A)	delA: >99.9%, A: <0.01%	delA: >99.9%, A: <0.01%
	*20	rs67666821(delA>A)	0.09%	0.09%
	*22	rs35599367 (C>T)	3.7%	2.6%
*CYP3A5*	*3	rs776746 (A>G)	92.4%	76.5%
	*6	rs10264272 (G>A)	<0.01%	0.2%

^#^ *UGT1A1* rs887829 was used as a surrogate predictor of UGT1A1*28. RefSeq reference sequences were used when available. ^$^ For variants not defining alleles, variant frequency is shown. For variants defining alleles, allele frequency is shown, except for *CYP2C8* and CYP3A4*2 and *6. Frequency information was obtained from https://www.ensembl.org/index.html, https://cpicpgx.org/ and https://www.ncbi.nlm.nih.gov/snp/ (accessed on 3 October 2023).

## Data Availability

Data belong to the clinical trials’ sponsors and may be accessible upon reasonable request to the corresponding authors.

## Supplementary Materials

The following supporting information can be downloaded at: https://www.mdpi.com/article/10.3390/ijms242015265/s1.
