# Supplementary material for: Impact of Sex and Genetic Variation in Relevant Pharmacogenes on the Pharmacokinetics and Safety of Valsartan, Olmesartan and Hydrochlorothiazide
Source: Int J Mol Sci. 2023 Oct 17;24(20):15265. doi: 10.3390/ijms242015265 (PMC10607223; doi:10.3390/ijms242015265)
Supplement: Supplementary file 1 [file ijms-24-15265-s001.zip › ijms-2626314-supplementary.pdf]

Impact of sex and genetic variation in relevant pharmacogenes on the pharmacokinetics and safety of valsartan, olmesartan and hydrochlorothiazide

Paula Soria-Chacartegui [1], Pablo Zubiaur [1,*], Dolores Ochoa [1], Marcos Navares-Gómez [1], Gonzalo Villapalos-García [1], Alejandro de Miguel [1], Eva González-Iglesias [1], Andrea Rodríguez-López [1], Gina Mejía-Abril [1], Samuel Martín-Vilchez [1], Sergio Luquero-Bueno [1], Manuel Román [1] and Francisco Abad-Santos [1,2*].

[1] Clinical Pharmacology Department, Hospital Universitario de La Princesa, Faculty of Medicine, Universidad Autónoma de Madrid (UAM), Instituto de Investigación Sanitaria La Princesa (IP), 28006 Madrid, Spain.

[2] Centro de Investigación Biomédica en Red de Enfermedades Hepáticas y Digestivas (CIBERehd), Instituto de Salud Carlos III, 28029 Madrid, Spain.

* Correspondence: pablo.zubiaur@uam.es (P.Z.), francisco.abad@uam.es (F.A.-S.).

SUPLEMMENTARY MATERIAL

Table S1. Hydrochlorothiazide pharmacokinetics regarding biogeographic origin, CT, genotypes and phenotypes.

Variable	n	AUC_{∞}/DW (ng*h*kg/ml*mg)	C_{max}/DW (ng*kg/ml*mg)	t_{max} (h)	$t_{1/2}$ (h)
**Biogeographic origin**					
European	37	3291.69 (709.97)	483.85 (111.12)	1.94 (0.70)	9.29 (1.55)
Other[#]	21	3398.60 (800.55)	461.39 (100.10)	1.98 (0.73)	9.28 (1.25)
**CT**					
2	4	3451.89 (372.63)	458.25 (51.09)	1.69 (0.38)	9.17 (1.64)
3	8	3002.88 (489.14)	424.38 (64.28)	2.16 (0.83)	9.21 (1.47)
4	8	2996.97 (206.41)	435.41 (85.73)	1.75 (0.87)	9.80 (1.21)
5	12	3658.72 (1122.00)	495.78 (121.43)	1.91 (0.78)	9.23 (1.12)
6	10	3704.02 (685.72)	562.78 (99.50)	2.18 (0.78)	9.35 (1.78)
7	16	3150.75 (612.93)	456.44 (112.63)	1.91 (0.53)	9.09 (1.61)
***ABCB1* rs1128503**					
T/T	15	3459.35 (1073.41)	481.41 (132.28)	2.10 (0.82)	9.15 (1.25)
C/T	21	3092.55 (450.55)	458.09 (97.68)	1.83 (0.44)	9.32 (1.58)
C/C	21	3485.00 (669.04)	494.09 (97.85)	1.89 (0.75)	9.40 (1.49)
***ABCB1* rs1045642**					
T/T	17	3307.74 (710.69)	452.87 (112.99)	2.09 (0.66)	9.29 (1.17)
C/T	21	3266.86 (917.42)	487.91 (109.53)	1.85 (0.64)	9.27 (1.63)
C/C	19	3430.70 (571.69)	488.01 (100.65)	1.85 (0.72)	9.36 (1.51)
***ABCB1* rs2032582**					
T/T	13	3194.44 (609.62)	445.81 (118.26)	1.98 (0.69)	9.58 (1.11)
T/G	20	3130.42 (847.09)	466.28 (113.20)	1.99 (0.72)	8.87 (1.64)
G/A+G/G+A/A	23	3540.31 (652.77)	495.42 (97.62)	1.88 (0.71)	9.56 (1.40)
***ABCC2* rs2273697**					
G/G	39	3370.66 (798.61)	482.11 (107.84)	1.95 (0.72)	9.33 (1.49)
G/G + G/A	17	3267.71 (643.01)	468.19 (110.84)	1.82 (0.57)	9.38 (1.30)
***ABCG2* rs2231142**					
NM	41	3350.88 (750.94)	483.59 (104.88)	1.94 (0.72)	9.31 (1.57)
IM	16	3289.55 (751.97)	461.87 (114.27)	1.86 (0.52)	9.29 (1.11)
***SLC22A1* rs72552763**					
GAT/GAT	37	3273.51 (776.85)	475.59 (105.66)	1.95 (0.72)	9.13 (1.57)
GAT/- + -/-	20	3444.95 (687.26)	481.01 (112.15)	1.87 (0.59)	9.63 (1.12)
***SLC22A1* rs12208357**					
C/C	53	3368.79 (756.78)	478.11 (107.96)	1.94 (0.67)	9.31 (1.45)
C/T	4	2868.32 (350.79)	469.28 (107.87)	1.63 (0.66)	9.27 (1.53)
**SLCO1B1**					
NF	45	3340.78 (803.52)	476.21 (110.95)	1.95 (0.68)	9.36 (1.39)
DF	10	3264.89 (518.28)	499.91 (86.98)	1.71 (0.68)	8.80 (1.69)
PF	2	3517.53 (371.95)	394.22 (112.23)	2.38 (0.18)	10.56 (0.22)
**Total**	58	3330.40 (738.86)	475.72 (106.92)	1.95 (0.71)	9.28 (1.44)

Data shown as mean (standard deviation). AUC_{∞}/DW: dose-weight corrected area under the curve. C_{max}/DW: dose-weight corrected maximum plasmatic concentration. $t_{1/2}$: half-life. CT:

clinical trial. NM: normal metabolizer. IM: intermediate metabolizer. NF: normal function. DF: decreased function. PF: poor function. #: individuals who self-reported as Latin-American or African.

Table S2. Valsartan pharmacokinetics regarding biogeographic origin, CT, genotypes and phenotypes.

Variable	n	AUC_∞/DW (ng*h*kg/ml*mg)	C_{max}/DW (ng*kg/ml*mg)	t_{max} (h)	$t_{1/2}$ (h)
**Biogeographic origin**					
European	17	11158.19 (4757.05)	1339.48 (465.41)	3.29 (0.93)	9.19 (2.30)
Other[#]	9	11367.40 (4038.32)	1463.42 (469.89)	3.92 (0.79)	7.80 (2.03)
**CT**					
1	6	8676.62 (3053.33)	1115.52 (367.98)	2.98 (0.63)	9.67 (3.33)
2	4	9598.88 (3000.41)	1241.25 (507.48)	3.47 (1.25)	8.43 (2.17)
3	8	13923.70 (5281.20)	1676.89 (418.49)	3.55 (0.92)	8.25 (2.12)
4	8	11268.88 (4072.81)	1358.60 (453.79)	3.88 (0.92)	8.58 (1.68)
***ABCC2* rs2273697**					
G/G	15	11729.36 (4400.82)	1485.70 (487.09)	3.50 (0.89)	8.26 (2.53)
G/G + G/A	10	10669.33 (4845.16)	1222.40 (419.29)	3.39 (0.97)	9.50 (1.77)
***ABCG2* rs2231142**					
NM	18	11534.59 (4616.63)	1400.01 (445.57)	3.34 (0.82)	8.95 (2.52)
IM	7	10715.86 (4536.14)	1329.92 (565.68)	3.77 (1.09)	8.26 (1.66)
**CYP2A6**					
*1/*1	22	11108.26 (4627.28)	1379.24 (483.78)	3.51 (0.93)	8.61 (2.36)
*1/*9	3	12750.69 (4024.65)	1388.72 (449.88)	3.04 (0.71)	9.81 (1.77)
**CYP2B6**					
RM	6	12201.51 (3059.67)	1533.82 (295.22)	3.59 (1.05)	7.66 (1.22)
NM	6	10269.17 (4302.27)	1214.73 (408.50)	4.00 (0.73)	8.22 (1.60)
IM	9	11613.57 (5744.72)	1384.08 (591.58)	3.16 (0.97)	9.47 (2.51)
PM	3	12948.58 (3038.92)	1618.05 (400.94)	3.34 (0.32)	10.33 (4.10)
**CYP2C8**					
RM	6	10392.32 (5530.99)	1203.76 (455.16)	2.80 (0.68)	8.99 (1.40)
NM	16	12030.36 (3954.15)	1471.71 (466.25)	3.71 (0.90)	9.04 (2.61)
IM	2	5787.45 (1918.53)	948.69 (341.29)	3.32 (1.15)	6.55 (1.31)
**CYP2C9**					
NM	15	10534.71 (3659.84)	1347.78 (433.15)	3.60 (0.79)	8.57 (2.69)
PM + IM	10	12461.30 (5571.47)	1429.29 (542.96)	3.25 (1.06)	9.03 (1.65)
***SLC22A1* rs72552763**					
GAT/GAT	15	10877.91 (4572.82)	1319.24 (437.64)	3.46 (0.83)	8.31 (1.71)
GAT/delGAT + delGAT/delGAT	10	11946.50 (4589.39)	1472.09 (526.86)	3.45 (1.05)	9.43 (2.96)
***SLC22A1* rs12208357**					
C/C	22	10999.14 (4651.83)	1341.92 (481.45)	3.47 (0.92)	8.89 (2.36)
C/T	3	13550.91 (2961.76)	1662.41 (300.82)	3.34 (0.97)	7.76 (1.79)
**SLCO1B1**					
NF	21	10583.92 (4341.31)	1309.98 (481.01)	3.47 (0.95)	9.01 (2.33)
PF + DF	4	15092.84 (3825.96)	1749.98 (118.10)	3.38 (0.69)	7.41 (1.81)
**UGT1A1**					
NF	10	10171.38 (4565.93)	1268.86 (454.36)	3.70 (0.99)	8.99 (2.77)
PF + DF	15	12061.33 (4472.96)	1454.73 (482.15)	3.29 (0.84)	8.60 (2.02)
**Total**	26	11230.61 (4439.80)	1382.38 (461.41)	3.51 (0.92)	8.71 (2.27)

Data shown as mean (standard deviation). AUC_∞/DW: dose-weight corrected area under the curve. C_{max}/DW: dose-weight corrected maximum plasmatic concentration. $t_{1/2}$: half-life. CT: clinical trial. RM: rapid metabolizer. NM: normal metabolizer. IM: intermediate metabolizer. PM: poor metabolizer. NF: normal function. DF: decreased function. PF: poor function. [#]: individuals who self-reported as Latin-American or African.

Table S3. Olmesartan pharmacokinetics regarding sex, biogeographic origin, genotypes and phenotypes.

Variable	n	AUC_∞/DW (ng*h*kg/ml*mg)	Cmax/DW (ng*kg/ml*mg)	t_{max} (h)	$t_{1/2}$ (h)
**Biogeographic origin**					
European	25	10910.12 (3027.80)	1448.44 (450.12)	2.62 (0.99)	9.06 (1.33)
Other#	12	10723.00 (3458.54)	1359.04 (348.72)	2.79 (1.16)	8.94 (1.48)
***ABCB1* rs1128503**					
T/T	12	12055.78 (4236.02)	1514.44 (550.57)	2.67 (1.09)	9.40 (1.25)
C/T	12	9749.10 (2215.46)	1315.71 (299.95)	2.33 (0.96)	9.00 (1.63)
C/C	13	10751.57 (2387.33)	1427.51 (376.70)	3.00 (1.02)	8.69 (1.18)
***ABCB1* rs1045642**					
T/T	12	11939.94 (4002.88)	1476.99 (543.19)	2.71 (1.16)	9.62 (1.16)
C/T	14	10298.28 (2831.47)	1434.90 (370.53)	2.36 (0.82)	8.91 (1.53)
C/C	11	10361.26 (2241.86)	1337.00 (334.20)	3.05 (1.11)	8.50 (1.18)
***ABCB1* rs2032582**					
T/T	9	11488.21 (3843.76)	1444.19 (585.37)	2.83 (1.23)	9.75 (1.12)
T/G	11	10349.10 (2629.67)	1394.23 (334.38)	2.41 (0.97)	8.99 (1.46)
G/G + A/A	15	10466.73 (2395.79)	1393.25 (360.68)	2.87 (1.03)	8.64 (1.40)
***ABCC2* rs2273697**					
G/G	28	10855.95 (3439.86)	1430.50 (448.96)	2.54 (1.08)	9.07 (1.42)
G/G + G/A	8	10838.89 (2151.04)	1384.80 (339.92)	3.06 (0.82)	8.99 (1.23)
***ABCG2* rs2231142**					
NM	28	10829.51 (2936.36)	1430.42 (403.54)	2.57 (1.06)	9.15 (1.42)
IM	9	10911.42 (3857.40)	1385.29 (481.47)	3.00 (0.94)	8.60 (1.11)
***SLC22A1* rs72552763**					
GAT/GAT	25	11212.22 (3325.80)	1474.58 (414.88)	2.82 (0.98)	8.99 (1.41)
GAT/-	12	10093.62 (2633.25)	1304.58 (415.58)	2.38 (1.13)	9.08 (1.30)
***SLC22A1* rs34059508**					
G/G	35	10737.38 (3107.81)	1396.54 (390.85)	2.74 (1.02)	9.01 (1.36)
G/A	2	12810.46 (3919.36)	1820.21 (855.32)	1.50 (0.00)	9.23 (1.97)
**SLCO1B1**					
NF	30	10440.45 (2858.62)	1358.67 (374.76)	2.80 (1.04)	9.10 (1.28)
PF + DF	7	12602.21 (3837.92)	1679.92 (517.46)	2.14 (0.90)	8.67 (1.72)
**Total**	37	10849.43 (3126.42)	1419.44 (417.17)	2.68 (1.04)	9.02 (1.36)

Data shown as mean (standard deviation). AUC_∞/DW: dose-weight corrected area under the curve. C_{max}/DW: dose-weight corrected maximum plasmatic concentration. $t_{1/2}$: half-life. NF: normal function. DF: decreased function. PF: poor function. #: individuals who self-reported as Latin-American or African.
